# Congenital hydrocephalus: a review of recent advances in genetic etiology and molecular mechanisms

**DOI:** 10.1186/s40779-024-00560-5

**Published:** 2024-08-12

**Authors:** Xiu-Yun Liu, Xin Song, Marek Czosnyka, Chiara Robba, Zofia Czosnyka, Jennifer Lee Summers, Hui-Jie Yu, Guo-Yi Gao, Peter Smielewski, Fang Guo, Mei-Jun Pang, Dong Ming

**Affiliations:** 1 Medical School, Tianjin University, Tianjin, 300072 China; 2 State Key Laboratory of Advanced Medical Materials and Devices, Tianjin University, Tianjin, 300072 China; 3 Haihe Laboratory of Brain-Computer Interaction and Human-Machine Integration, Tianjin, 300380 China; 4 Department of Clinical Neurosciences, Addenbrooke’s Hospital, University of Cambridge, Cambridge, CB2 0QQ UK; 5 San Martino Policlinico Hospital, IRCCS for Oncology and Neuroscience, 16132 Genoa, Italy; 6 Department of Anesthesiology and Critical Care Medicine, Johns Hopkins University, Baltimore, MD 21287 USA; 7 Department of Neurosurgery, Tianjin Medical University General Hospital, Tianjin, 300052 China; 8 Department of Neurosurgery, Beijing Tiantan Hospital, Capital Medical University, Beijing, 100070 China; 9 Department of Neurosurgery, Tianjin Huanhu Hospital, Tianjin, 300350 China; 10 School of Pharmaceutical Science and Technology, Tianjin University, 300072 Tianjin, China

**Keywords:** Congenital hydrocephalus, Genetic disease, Central system, Cilia, Ventricle

## Abstract

The global prevalence rate for congenital hydrocephalus (CH) is approximately one out of every five hundred births with multifaceted predisposing factors at play. Genetic influences stand as a major contributor to CH pathogenesis, and epidemiological evidence suggests their involvement in up to 40% of all cases observed globally. Knowledge about an individual’s genetic susceptibility can significantly improve prognostic precision while aiding clinical decision-making processes. However, the precise genetic etiology has only been pinpointed in fewer than 5% of human instances. More occurrences of CH cases are required for comprehensive gene sequencing aimed at uncovering additional potential genetic loci. A deeper comprehension of its underlying genetics may offer invaluable insights into the molecular and cellular basis of this brain disorder. This review provides a summary of pertinent genes identified through gene sequencing technologies in humans, in addition to the 4 genes currently associated with CH (two X-linked genes *L1CAM* and *AP1S2*, two autosomal recessive *MPDZ* and *CCDC88C*). Others predominantly participate in aqueduct abnormalities, ciliary movement, and nervous system development. The prospective CH-related genes revealed through animal model gene-editing techniques are further outlined, focusing mainly on 4 pathways, namely cilia synthesis and movement, ion channels and transportation, Reissner’s fiber (RF) synthesis, cell apoptosis, and neurogenesis. Notably, the proper functioning of motile cilia provides significant impulsion for cerebrospinal fluid (CSF) circulation within the brain ventricles while mutations in cilia-related genes constitute a primary cause underlying this condition. So far, only a limited number of CH-associated genes have been identified in humans. The integration of genotype and phenotype for disease diagnosis represents a new trend in the medical field. Animal models provide insights into the pathogenesis of CH and contribute to our understanding of its association with related complications, such as renal cysts, scoliosis, and cardiomyopathy, as these genes may also play a role in the development of these diseases. Genes discovered in animals present potential targets for new treatments but require further validation through future human studies.

## Background

Congenital hydrocephalus (CH) is characterized by the excessive accumulation of cerebrospinal fluid (CSF) in the brain at birth [1]. The incidence of CH is approximately 1/500 among young individuals and 2/500 among the elderly. It is a complex brain disorder with multiple etiological factors, including vitamin B or folic acid deficiency, intraventricular hemorrhage, viral infections, environmental influences, developmental anomalies, and genetic predisposition, often accompanied by structural brain abnormalities and neural dysfunction [2]. Common symptoms of hydrocephalus include gait disturbances, cognitive impairment, urinary dysfunction, seizures, abnormal reflexes, bradycardia and hypoventilation, headaches, vomiting, and visual impairments [3]. Among these factors contributing to CH development, global epidemiological data suggests that genetic factors account for more than 40% of cases [4, 5]. The annual medical costs associated with hydrocephalus are estimated at around $2 billion per year in the US alone, thus posing a significant economic and societal burden [6].

Though genetic factors contribute to up to 40% of cases of CH, precise genetic causes have only been identified in less than 5% of human cases [4]. There is a pressing need for a deeper understanding of the genetic components and mechanisms underlying CH, which has the potential to yield invaluable insights into its molecular and cellular etiology [7]. This review aims to consolidate existing evidence on the pathologic genes implicated in both human patients and animal models with respect to CH development. The goal is to stimulate novel approaches towards treating CH. Additionally, we discuss other developmental disorders and organ dysfunctions associated with genes related to hydrocephalus.

## The production and circulation of CSF

CSF plays a critical role not only in providing mechanical support for the brain and spinal cord but also serves as a carrier for transporting metabolic waste and nutrients [8]. The healthy brain consists of three integrated components that collectively regulate CSF dynamics: CSF production, circulation, and absorption. These three components typically maintain equilibrium.

**Fig. 1:**
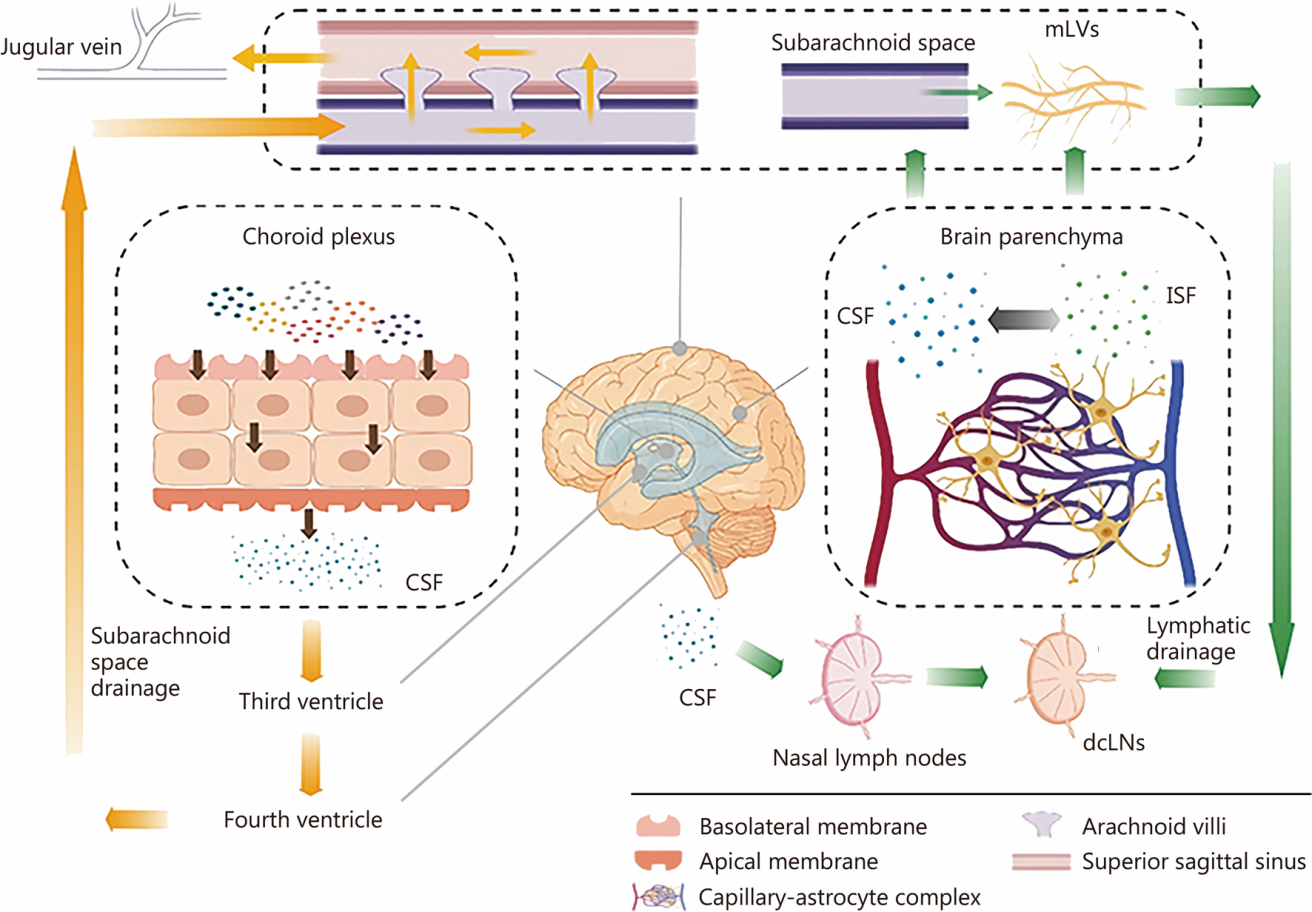
The production of CSF occurs through two distinct pathways: the choroid plexus and the brain parenchymal system. CSF can be absorbed by the subarachnoid space or glymphatic circulation, ultimately entering the dcLNs. CSF cerebrospinal fluid, dcLNs deep cervical lymphatic nodes, ISF interstitial fluid, mLVs meningeal lymphatic vessels

Approximately 80–90% of CSF is produced by the choroid plexus in the cerebral lateral ventricles [9, 10] (Fig. 1). Ion transporters on the basolateral membrane facing the blood and the apical membrane facing the ventricles are responsible for secreting and delivering ions such as Na^+^, Cl^−^ and HCO_3_^−^ from the blood to the ventricles [11–13]. The remaining 10–20% of CSF production is attributed to the brain parenchymal system through exchange between CSF and interstitial fluid (ISF) in the capillary-astrocyte complex.

Most researchers have hypothesized that the circulation of CSF commences from the lateral ventricles, proceeds into the third ventricle, and then passes into the fourth ventricle through the midbrain cerebral aqueduct. The majority of CSF subsequently flows into the cisterna magna and cerebellopontine cisterns via the apertures of the fourth ventricle, namely, the median aperture and two lateral apertures. Ultimately, it is reabsorbed into the cerebral venous system through the arachnoid villi [14]. The extracranial lymphatic drainage pathway serves as a crucial component of CSF circulation, playing a pivotal role in maintaining homeostasis, buffering functions, and protective mechanisms of the central nervous system (CNS) [15]. As illustrated in Fig. 1, a significant volume of CSF drains into nasal lymph nodes and meningeal lymphatic vessels (mLVs), through which CSF is removed from intracranial spaces to extracranial regions and subsequently absorbed by the deep cervical lymphatic nodes (dcLNs) [16]. This intricate physiological process involves interactions among multiple molecules. Therefore, in subsequent sections, we will focus on pathological mechanisms related to molecular dysfunctions causing hydrocephalus. Disruption in any of these processes could lead to excessive accumulation of CSF and ventriculomegaly due to factors such as CSF overproduction, inefficient reabsorption into the systemic circulation, abnormal cilium-dependent flow, or obstruction within the ventricular system.

## The main genetic target of CH in humans

**Fig. 2:**
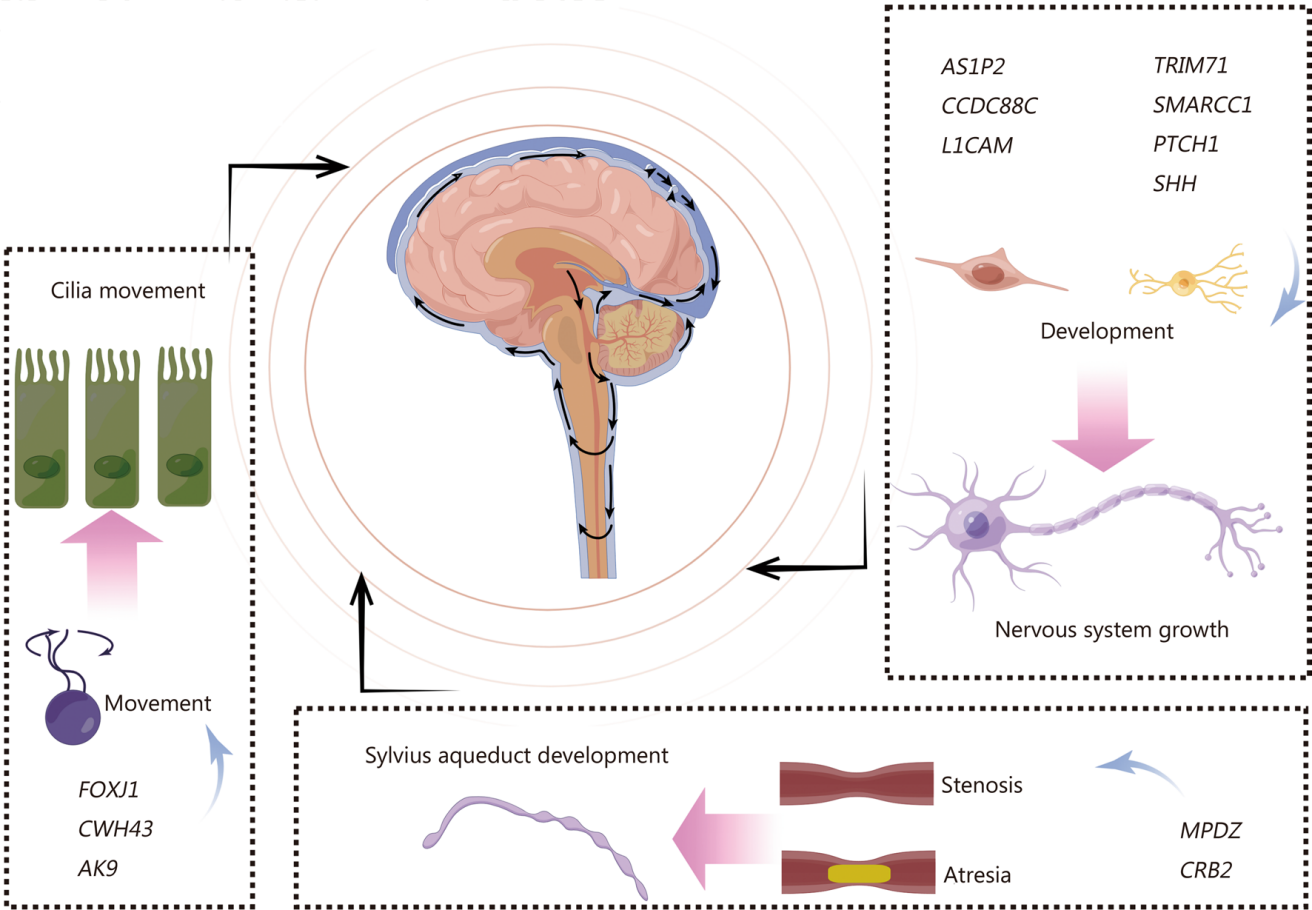
The genetic targets of CH in humans involve genes related to cilia movement, Sylvius aqueduct development, and nervous system growth in pathological cases of CH. CH congenital hydrocephalus, FOXJ1 forkhead box J1, CWH43 cell wall biogenesis 43 C-terminal homolog, AK9 adenylate kinase 9, AP1S2 adaptor related protein complex 1 subunit sigma 2, CCDC88C coiled-coil domain containing 88C, L1CAM L1 cell adhesion molecule, TR1M71 tripartite motif containing 71, SMARCC1 SWI/SNF related, matrix associated, actin dependent regulator of chromatin subfamily C member 1, PTCH1 patched 1, SHH sonic hedgehog, MPDZ multiple PDZ domain crumbs cell polarity complex component, CRB2 crumbs cell polarity complex component 2

Genes associated with CH in human cases are presented in Fig. 2, most of which are involved in Sylvius aqueduct (SA) defects, cilia growth and movement, and nervous system development. The Human Phenotype Ontology website predicts that 411 genes are related to “hydrocephalus” (HP:0000238). Among them, only 4 genes have been confirmed to be linked to CH: two X-linked genes [*L1CAM* (L1 cell adhesion molecule) and *AP1S2* (adaptor-related protein complex 1 subunit sigma 2)] and two autosomal recessive genes [*MPDZ* (multiple PDZ domain crumbs cell polarity complex component) and *CCDC88C* (coiled-coil domain containing 88C)].

SA stenosis, which connects the third and fourth ventricles, is responsible for the majority of cases of non-syndromic CH. Approximately 5–15% of cases are associated with X-linked variations of *L1CAM*, known as L1 syndrome. *L1CAM* encodes a transmembrane glycoprotein belonging to the immunoglobulin superfamily of cell adhesion molecules, and it plays important roles in neuronal adhesion, migration, growth cone morphology, neurite outgrowth, and myelination. Another separate X-linked syndrome called Fried-Pettigrew syndrome [Online Mendelian Inheritance in Man (OMIM): 304,340], is characterized primarily by intellectual disability, basal ganglia iron or calcium deposition, and hydrocephalus due to *AP1S2* variation [17–19]. Variations in *MPDZ* and *CCDC88C* share many neuropathological similarities including atresia of both SA and the central canal of the medulla with recessive forms of CH (OMIM: 615,219 and OMIM: 236,600 respectively). Both genes colocalize at the apical cell junction in the neural plate, *CCDC88C* directly interacts with *MPDZ* and cooperates to promote apical cell constriction during neurulation [20, 21]. *MPDZ* is essential for maintaining ependymal integrity, loss of *MPDZ* leads to ependymal denudation accompanied by reactive astrogliosis and SA stenosis [22]. Additionally, mutations in *MPDZ* can cause abnormally high permeability in choroid plexus epithelial cell monolayers [23].

Moreover, this section also provides a summary of the mutation genes identified through gene sequencing technology in cases of hydrocephalus and related diseases, which require further validation to establish their causal involvement in hydrocephalus. Regarding SA development-related genes, *CRB2* encodes the crumbs cell polarity complex component 2, originally primarily associated with renal anomalies such as renal tubular or glomerular microcysts. Recently, Tessier et al. [24] reported that biallelic *CRB2* variations are also strongly linked to hydrocephalus, resulting from atresia of the SA and central canal aqueduct of the medulla.

For the genes related to cilia growth and motility, *CWH43* (cell wall biogenesis 43 C-terminal homolog) is highly expressed in ciliated ependymal and choroid plexus cells, where it regulates the membrane localization of glucose-6-phosphate isomerase (GPI)‐anchored proteins in mammalian cells. Yang et al. [25] found that approximately 15% of patients with idiopathic normal pressure hydrocephalus (iNPH) carry heterozygous loss-of-function deletions in *CWH43*. Similarly, mice with *Cwh43* deletions could develop communicating hydrocephalus, gait dysfunction, and abnormalities in choroid plexus and ependymal cells. The mutation of *CWH43* affects the number of ependymal cilia and the apical/basal targeting of GPI‐anchored proteins in ventricular multi-ciliated epithelial cells, which may contribute to the development of iNPH. *AK9*, encoding adenylate kinase 9, was also suggested to be involved in iNPH. A damaging mutation in *AK9* was detected in 9.6% of iNPH patients [26]. Mice with *Ak9* mutation exhibit decreased cilia motility and beat frequency, as a result of communicating hydrocephalus and balance impairment. Dysfunction of the *FOXJ1* (forkhead box J1) triggers autosomal dominant motile ciliopathies affecting many organ systems, including brain ventricles leading mainly to abnormal ventricular ciliary motility in CH [27]. *CC2D2A* (coiled-coil and C2 domain containing 2A) mutations are a relatively common cause of Joubert syndrome, a ciliopathy characterized by distinctive brain malformation and developmental delay. Patients with *CC2D2A* mutations often present with hydrocephalus or epilepsy [28]. Furthermore, Munch et al. [29] investigation revealed that 14 genes are involved in ciliogenesis, *CELSR2* (cadherin EGF LAG seven-pass G-type receptor 2), *CENPF* (centromere protein F), *DNAI1* (dynein axonemal intermediate chain 1), *DNAH5* (dynein axonemal heavy chain 5), *FLNA* (filamin A), *FUZ* (fuzzy planar cell polarity protein), *IFT172* (intraflagellar transport 172), *LRP6* (LDL receptor-related protein 6), *MPDZ*, *NOTCH2* (Notch receptor 2), *PIK3R2* (phosphoinositide-3-kinase regulatory subunit 2), *PTCH1* (patched 1), *TRIM71* (tripartite motif containing 71), and *VANGL2* (VANGL planar cell polarity protein 2).

In relation to the nervous system’s function, *TRIM71*, *SMARCC1* (SWI/SNF related, matrix-associated, actin-dependent regulator of chromatin subfamily C member 1), *PTCH1*, and *SHH* (sonic hedgehog) play crucial roles in both neural tube development as well as neural stem cell (NSC) growth. Furey et al. [30] identified mutations within these aforementioned 4 genes among 125 CH trios and 52 independent probands through whole exome sequencing (WES). *SMARCC1* encodes for SWI/SNF-related, matrix-associated, actin-dependent regulator of chromatin, subfamily C, member 1 (BAF155) which is a chromatin remodeling protein, its mutation results in CH phenotype associated with defects during neural tube development [31–33]. Additionally, 6 other genes, *ASTN2* (astrotactin 2), *B3GALNT2* (beta-1,3-N-acetylgalactosaminyltransferase 2), *DAG1* (dystroglycan 1), *NF1* (neurofibromin 1), *ROBO1* (roundabout guidance receptor 1), and *SMARCC1* participate in processes related to neuronal formation [29].

In addition, several genes have been identified as being related to hydrocephalus, but the reporting of this relationship has been incomplete. *MMACHC* (metabolism of cobalamin associated C) mutation with c.609G > A is most frequently observed in patients with cobalamin C deficiency (cblC). Recent research has shown that the homologous mutation *MMACHC* c.609G > A often leads to irreversible brain disorders such as developmental delay, seizures, and hydrocephalus [34]. Furthermore, a study of 27 CH families revealed that the *WDR81* (WD repeat domain 81) and *EML1* (EMAP like 1) genes are associated with CH [35]. Another study involving 381 sporadic CH cases (232 trios) identified several new risk genes of CH including *PIK3CA* (phosphatidylinositol-4,5-bisphosphate 3-kinase catalytic subunit alpha), *PTEN* (phosphatase and tensin homolog), *mTOR* (mechanistic target of rapamycin kinase), *FMN2* (formin 2), and *FXYD2* (FXYD domain-containing ion transport regulator 2) [19]. Additionally, a study of 110 infantile hydrocephalus cases indicated that *ZEB1* (zinc finger E-box binding homeobox 1), *SBF2* (SET binding factor 2), and *GNAI2* (G protein subunit alpha i2) were over-represented and might affect the signaling pathways involved in infantile hydrocephalus formation [36].

Overall, due to limited data and research, the current findings can only account for less than 5% of primary CH cases [37]. Further genome sequencing of large, well-phenotype cohorts is necessary to gain a deeper understanding of the molecular and cellular etiology of CH.

## The main genetic targets of CH in animal models

**Fig. 3:**
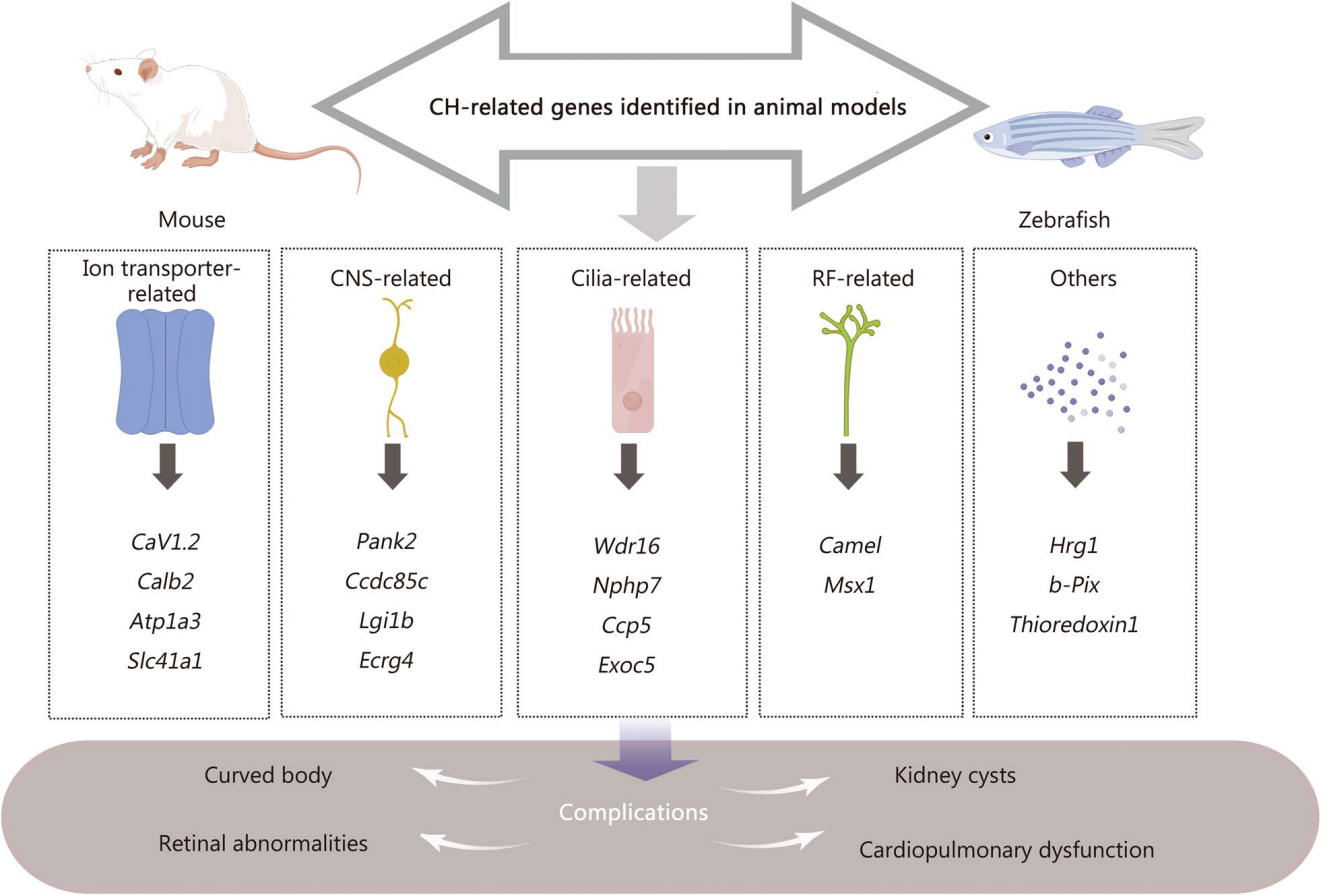
Genes associated with CH identified in animal models. In zebrafish and mouse models, genes linked to the development of hydrocephalus can be categorized into 4 distinct groups: cilia synthesis and movement-related, ion transporter-related, RF synthesis-related, cell apoptosis and neurogenesis-related, etc. RF Reissner’s fiber, CH congenital hydrocephalus, CNS central nervous system, CaV1.2 calcium voltage-gated channel subunit alpha1 C, Calb2 calbindin 2, Atp1a3 ATPase Na^+^/K^+^ transporting subunit α3, Slc41a1 solute carrier family 41 members 1, Pank2 pantothenate kinase 2, Ccdc85c coiled-coil domain containing 85C, Lgi1b leucine-rich glioma inactivated 1b, Ecrg4 esophageal cancer related gene 4, Wdr16 cilia and flagella associated protein 52, Nphp7 nephrocystin 7, Ccp5 cytosolic carboxypeptidases 5, Exoc5 cxocyst complex component 5, Msx1 Msh homeobox 1, Hrg1 solute carrier family 48 member 1, b-Pix Rho guanine nucleotide exchange factor (GEF) 7b

Animal models of CH exhibit numerous histopathological similarities to humans, making them valuable for studying the genetics and pathogenesis of CH. Many genetic loci associated with hydrocephalus have been identified in animal models [38]. In this section, we provide a summary of CH-related genes discovered in animal models, most of which are related to cilia synthesis and movement, ion transportation, RF synthesis, cell apoptosis, and neurogenesis (Fig. 3).

### Cilia-related genes

Ciliated structures composed of microtubules form elongated protrusions on cellular membranes, they can be found in various cell types including ependymal cells. Cilia can be classified into two categories: primary cilium which serves primarily as a sensor for signal transduction [39], and motile cilium is found predominantly on specialized cells responsible for fluid movement or cell propulsion through outer dynein arms (ODA) and inner dynein arms (IDA) [40]. Malfunctioning ciliary activity may lead to genetic developmental disorders associated with primary ciliary dyskinesia (PCD), leading to conditions such as infertility, developmental anomalies, hydrocephalus, and auditory issues along with compromised respiratory pathogens clearance leading susceptibility towards infections causing persistent coughing and dyspnea [41]. Ependymal cells are located in the superficial layer of the cerebral ventricle walls and the central canal of the spinal cord. The cilia on these cells play a role in producing and circulating CSF as well as contributing to nerve regeneration. Both primary and motile cilia are involved in hydrocephalus through distinct mechanisms related to their physiological functions. Cilia distributed in various regions of the ventricles work together to maintain the directional flow of CSF. Growing evidence indicated that coordinated beating of motile cilia generates significant force, propelling CSF production and circulation within brain ventricles [42, 43]. Impairment of ciliary motor function can disrupt the balance between CSF production and circulation, resulting in the accumulation of CSF in the ventricles. Table 1 presents a list of 28 genes that regulate the structure and function of cilia [44–74].

**Table 1:** List of potential CH-related genes in animal models

Category	Gene	Function description	Relationship with CH	Other related diseases (in addition to hydrocephalus)	Gene manipulation method	Species	References
Cilia-related	*Wdr16*	Regulate cilia signal transduction	Affect cell polarity such as water homeostasis or osmoregulation to induce hydrocephalus, but ependymal disorganization or impaired ciliary motility was not observed	Anatomical structure abnormalities	Morpholino	Zebrafish	[44, 45]
	*Wdr78*	Encode motile cilium- specific protein and is involved in axon-dynein complex assembly and ciliary movement	Affect cilia assembly and movement	Abnormal otolith, pronephric cysts	Morpholino	Zebrafish	[46]
	*Nphp7*	Encode the Kruppel-like zincfinger transcription factor GLIS2 and has an interaction with BBS1	Disrupt cilia beat	Cystic pronephros	Morpholino	Zebrafish	[47]
	*Ccp5*	Encode a metal carboxypeptidase belonging to the M14 peptidase family and involved in the process of post-translational modification of tubulin	Uncoordinated movement and reduce the amplitude of cilia	Ventral body curvature, pronephric cysts	Morpholino	Zebrafish	[48, 49]
	*Exoc5*	Encode a protein component of the exocyst complex and play a crucial role in ciliogenesis	Affect ciliogenesis	Cardiac edema	Mutation	Zebrafish	[50]
	*Efcab1*	Encode Ca^2+^-binding dynein-associated protein and serve as a crucial regulator in coordinating ciliary movement	Block cilia movement and impair CSF flow	PCD phenotypes like situs inversus	Mutation	Mice	[51]
	*Adamts9*	A substrate for B3glct, encode secreted extracellular metalloproteinase and is critical in the formation and maintenance of primary cilia's functional integrity	Mediate cilia shorten	Ciliopathic phenotypes like renal cysts	Morpholino	Zebrafish	[52]
	*B3glct*	β3-glucosyltransferase	Reduce cilia basal bodies and changes inner epithelium polarity; and reduce secretion of SSPO	Skeletal abnormalities, white spotting	Mutation	Mice	[53]
	*Dyx1c1*	Encode a dynein protein axon assembly factor and is critical to ciliary assembly and functional integrity	Affect IDA and ODA assembly and ciliary dyskinesia	Body curvature, situs inversus, and kidney cysts	Morpholino	Zebrafish	[54]
	*Zmynd10*	Encode a cytoplasmic protein in cilia	Affect IDA and ODA assembly	PCD-like laterality defects	Mutation	Mice	[55]
	*Chmp4b*	Encode a protein in the CHMP family, and is a subunit of the endosomal sorting complexes required for transport	Affect cilia assembly	Curved body axis, otolith malformation, and kidney cyst	Morpholino	Zebrafish	[56]
	*Ttc30*	Involve in protein polyglutamylation in cilia and axonal microtubules	Affect ciliogenesis	Kidney cysts, left–right asymmetry	Mutation	Zebrafish	[57]
	*Dnaaf3*	Encode a cytoplasmic factor and is essential for the preassembly of axonemal IDA and ODA	Affect IDA and ODA assembly	PCD-like axis curvature defects, kidney cysts, perturbed otolith development, and laterality defects	Morpholino	Zebrafish	[58]
	*Nphp3*	Encode a protein with a coiled-coil domain, a tubulin-tyrosine ligase domain, and a tetratrico peptide repeat domain	Reduce cilia length and number	Pronephric cysts	Morpholino	Zebrafish	[59]
	*Wtip*	Encode an LIM domain protein of the Ajuba/Zyxin family and is involved in cell mitosis	Affect the PCP	Pronephric cysts, cloaca malformation, body curvature, and pericardial edema	Morpholino	Zebrafish	[60]
	*Ptk7*	Encode a protein that regulates signaling at the cell membrane and is critical for cell polarity and adhesion	Affects EC cilia function	Idiopathic scoliosis	Mutation	Zebrafish	[61]
	*Pard3*	Encode a polarity protein that cooperates with microtubules to regulate cell polarity	Impair ciliary growth	–	Morpholino	Zebrafish	[62]
	*Nherf1*	Encode a protein of the PDZ family and is abundantly expressed in ependymal epithelial cells	Impair cilia function	Renal dysfunction	Mutation/morpholino	Mice/Zebrafish	[63, 64]
	*Cep120*	Encode a protein involved in coupling centrosome microtubules, exhibiting a significant role in ciliogenesis	Affect ciliogenesis	Cerebellar hypoplasia/ventrally curved body axis, otolith defects, and cardiac edema	Mutation/Morpholino	Mice/Zebrafish	[65]
	*Pkd1 & Pkd2*	Encode polycystin, a kind of large membrane proteins that form a receptor-channel complex expressed throughout the body, and its expression in ependymal cells and choroid plexus	Affect cilia function	Dorsal axis curvature, pronephric cyst/nephric cyst	Morpholino/conditional inactivation or knockout	Mice/Zebrafish	[66, 67]
	*Pde1a*	Encode cAMP or cGMP hydrolyzed protein is activated by the Ca^2+^-calmodulin complex and plays an important role in signal transduction	Affect ciliary movement	Pronephric cysts, body curvature	Morpholino	Zebrafish	[68]
	*Pih1d3*	An X-linked gene is associated with the defects of dynein arms	Regulating the integrity structure and function of IDA and ODA	Situs inversus, defects in spermatocyte survival and mucociliary clearance	TALEN	Rats	[69]
	*Snx27*	Participate in the trafficking of multiple transmembrane receptors; Regular β-amyloid and Notch intracellular domain generation	Reduce ependymal cells and cilia density	–	Mutation	Mice	[70]
	*Cfap54*	Localize to the C1D projection of the central microtubule apparatus	Decrease the ciliary beat frequency and perturb cilia-driven flow	PCD-relevant phenotypes	Knockout	Mice	[71]
	*Ccdc39*	Is essential for the dynein motor protein regulatory complex	Disturb the axonemal organization and ciliary beating	–	Mutation	Mice	[72]
	*Stk36*	Encode a kind of serine-threonine protein kinases of the Unc-51–like kinase family	Affect the length and ultrastructure of the cilia	Respiratory infections	Knockout	Mice	[73]
	*Spag6l*	A key component of the central apparatus	Reduced ciliary beating frequency or uncoordinated beating	Growth retardation, structural abnormalities in the spleen	Conditional knockout	Mice	[74]
Ion transport-related	*CaV1.2*	Encode the Ca^2+^channel protein α-1 subunit for regulating Ca^2+^ transportation	Destroy the balance of cellular homeostasis	Primary cilia defects related diseases like renal cysts, left–right asymmetry defects	Morpholino	Zebrafish	[75]
	*Calb2a & Calb2b*	Encode a member of the troponin C superfamily of Ca^2+^ binding protein plays a role in Ca^2+^ transportation	Destroy nervous system development by regulating calcium concentration of synaptic	Axial curvature defect, and yolk sac edema	Morpholino	Zebrafish	[76]
	*Tmem67*	Regulate choroid plexus epithelial cell fluid and electrolyte homeostasis	Osmotic gradient increase in the choroid plexus epithelial cells	Polycystic kidney disease	Mutation	Rats	[77]
	*Atp1a3*	Encode a crucial enzyme responsible for transporting ions across membranes and regulate Na^+^ and K^+^ gradients, and is essential for the transmission of electrical excitation in both nerves and muscles	Destroy transmembrane ion transport	Neuronal excitability impaired	Morpholino	Zebrafish	[78]
	*Slc41a1*	Encode Mg^2+^ transporter a protein located in the basolateral plasma membrane and involved in the transmembrane transportation of Mg^2+^	Perturb intracellular Mg^2+^ homeostasis	Body curvature, cystic kidneys	Morpholino	Zebrafish	[79]
CNS-related	*Pank2*	Encode a key regulatory enzyme in the biosynthesis of coenzyme A	Impair neuronal development, particularly in the anterior part of the CNS	Perturbed brain morphology, heart region, and caudal plexus edema	Morpholino	Zebrafish	[95]
	*Ecrg4*	Encode auguring protein, is expressed in the CP epithelial, brain ventricular and central canal cells of the spinal cord	Inhibit CNS injury	–	Morpholino	Zebrafish	[96]
	*Ccdc85c*	Encode a protein belonging to the delta-interacting protein A family, and plays a necessary role in epithelial cell proliferation and cortex development	Reduce NPC number	Subcortical heterotopia, intracranial hemorrhage	Mutation	Mice	[97]
	*α-Snap*	Encode a key protein in intracellular trafficking	Result in abnormal transport of N-cadherin to the plasma membrane of NSCs	Abnormal neurogenesis	Mutation	Mice	[82]
	*Smarcb1*	The core subunit of the BAF chromatin remodeling complex is essential for the regulation of DNA accessibility and gene expression during neuronal differentiation	Affect neurodevelopment	Neuronal signaling disturbance	Mutation	Mice	[98]
	*Idh1*	Located in the cytoplasm and peroxisome where it acts in lipid and glucose metabolism and protects against ROS	SVZ cells proliferate ectopically, infiltrate the brain parenchyma, and form nodules; Self-renewal and proliferation of NSCs and NPCs increase	Grossly dilated lateral ventricles and gliomagenesis	Mutation	Mice	[99]
	*Lgi1b*	Encode a kind of secreted protein in the leucine-rich repeat (LRR) superfamily, it is highly expressed in the choroid plexus and involved in neuronal growth and survival	Mediate neuronal apoptosis	Brain dysplasia and pericardial edema	Morpholino	Zebrafish	[100]
RF-related	*Camel*	A novel distantly related member of the L1CAM family and involved in cell adhesion	Affect RF synthesis; abnormal development of CVOs and axial structures	Scoliosis (tail curled down)	Morpholino	Zebrafish	[105]
	*Msx1*	Regulate the activity of DNA-binding transcription factor is widely expressed in neuroepithelial cells, such as the fimbria and the medulla	Affect RF synthesis	–	Mutation	Mice	[110]
Others	*Hrg1*	Encode transmembrane protein, play an essential role in the formation and maturation of the erythrocytes	Affect heme homeostasis	Yolk tube malformations, anemia	Morpholino	Zebrafish	[111]
	*β-Pix*	Expressed in the brain and blood vessels	Disruption of vascular stability	Cranial hemorrhage	Mutation and morpholino	Zebrafish	[112]
	*Dio3*	Inactivate 2 main thyroid hormones and reduce the levels of THs during the early development of mammals	Affect the development	Cleft palate, choanal atresia, Chiari malformations	Mutation	Mice	[113]
	*Mks1*	Encode a 559 amino acid B9-domain-containing protein that localized to the basal body of mammalian cells	Wnt/β-catenin signal increase and cellular over-proliferation	Dandy-Walker malformation and renal cystic dysplasia	Knockout	Mice	[114]
	*Thioredoxin1*	Encode antioxidant protein	Mediate ventricular epithelial cell apoptosis	Brain dysplasia	Morpholino	Zebrafish	[115]

*CH* congenital hydrocephalus, *CSF* cerebrospinal fluid, *IDA* inner dynein arms, *ODA* outer dynein arms, *PCD* primary ciliary dyskinesia, *PCP* planar cell polarity, *EC* ependymal cell, *CNS* central nervous system, *CP* choroid plexus, *NPC* neural progenitor cell, *NSC* neural stem cell, *ROS* reactive oxygen species, *SVZ* subventricular zone, *CVO* circumventricular organ, *RF* Reissner’s fiber, *Wdr16* cilia and flagella associated protein 52, *Wdr78* dynein axonemal intermediate chain 4, *Nphp7* nephrocystin 7, *Ccp5* cytosolic carboxypeptidases 5, *Exoc5* cxocyst complex component 5, *Efcab1* EF-hand calcium binding domain 1, *Adamts9* ADAM metallopeptidase with thrombospondin type 1 motif 9, *B3glct* β3-glucosyltransferase, *Dyx1c1* dynein axonemal assembly factor 4, *Zmynd10* zinc finger MYND-type-containing 10, *Chmp4b* charged multivesicular body protein 4b, *Ttc30* tetratricopeptide repeat domain 30, *Dnaaf3* dynein axonemal assembly factor 3, *Nphp3* nephrocystin 3, *Wtip* WT1 interacting protein, *Ptk7* protein tyrosine kinase 7, *Pard3* Par-3 family cell polarity regulator, *Nherf1* Na^+^/H^+^ exchanger regulatory factor-1, *Cep120* centrosomal protein 120, *Pkd1* polycystic kidney disease 1, *Pde1a* Phosphodiesterase 1a, *Pih1d3* dynein axonemal assembly factor 6, *Snx27* sorting nexin 27, *Cfap54* cilia and flagella associated protein 54, *Ccdc39* coiled-coil domain 39 molecular ruler complex subunit, *Stk36* serine/threonine kinase 36, *Spag6l* sperm associated antigen 6-like, *CaV1.2* calcium voltage-gated channel subunit alpha1 C, *Calb2a* calbindin 2a, *Tmem67* transmembrane protein 67, *Atp1a3* ATPase Na^+^/K^+^ transporting subunit α3, *Slc41a1* solute carrier family 41 member 1, *Pank2* pantothenate kinase 2, *Ecrg4* esophageal cancer related gene 4, *Ccdc85c* coiled-coil domain containing 85C, *α-Snap* NSF attachment protein alpha, *Smarcb1* SWI/SNF related, matrix associated, actin dependent regulator of chromatin, subfamily b, member 1, *Idh1* isocitrate dehydrogenase (NADP^+^) 1, *Lgi1b* leucine rich glioma inactivated 1b, *Msx1* Msh homeobox 1, *Hrg1* solute carrier family 48 member 1, *β-Pix* Rho guanine nucleotide exchange factor (GEF) 7b, *Dio3* iodothyronine deiodinase 3, *Mks1* MKS transition zone complex subunit 1, *GLIS2* GLIS family zinc finger 2, *BBS1* Bardet-Biedl syndrome 1, *CHMP* charged multivesicular body protein, *SSPO* SCO-spondin, *cAMP* cyclic adenosine monophosphate, *cGMP* cyclic guanosine monophosphate, *TALEN* transcription activator-like effector nuclease, *L1CAM* L1 cell adhesion molecule, *THs* thyroid hormones

*Wdr16* (cilia and flagella-associated protein 52) plays a crucial role in cilia-related signal transduction. In zebrafish, severe hydrocephalus was observed in the *Wdr16* gene knockdown zebrafish. It is noteworthy that hydrocephalus was the phenotype of *Wdr16* disruption in zebrafish, but ependymal disorganization or impaired ciliary motility was not observed [44, 45]. It’s speculated that *Wdr16* regulates hydrocephalus through cilia-mediated cell polarity effects such as water homeostasis or osmoregulation. *Wdr78* (dynein axonemal intermediate chain 4) encodes a motile cilium-specific protein involved in the assembly of the axon dynein complex and ciliary movement. Depletion of *Wdr78* in mice caused defects in ependymal cilia, while *Wdr78* morphants zebrafish exhibited ciliopathy-associated phenotypes such as hydrocephalus, pronephric cysts, or abnormal otoliths [46]. Therefore, studies have shown that depletion of *Wdr78* leads to abnormal ciliary beat function of ectodermal cells by affecting the dynein-f assembly. *Nphp7* (nephrocystin 7) is a type of transcription factor and has been found to physically interact with Bardet-Biedl syndrome 1 (BBS1). A previous study indicated that hydrocephalus and pronephric cysts were displayed in the *Nphp7* zebrafish morphants [47]. It is noteworthy that the deletion of *Nphp7* revealed an astonishingly impaired ciliary motility.

### Ion channels and ion transporter-related genes

Ion transporters play important roles in the process of CSF secretion. Due to the unidirectional nature of ion movement, transporters located on the basement membrane side differ from those on the apical membrane side. These transporters effectively maintain internal homeostasis and balance of Na^+^, Cl^−^, and HCO_3_^−^, which in turn regulate CSF secretion. In this section, we examine 6 genes associated with ion transporter function, whose dysfunction could impact CSF secretion and lead to hydrocephalus (Table 1) [75–79].

*Calb2* (calbindin 2) belongs to the troponin C superfamily of Ca^2+^ binding protein and is involved in Ca^2+^ transportation. In zebrafish, C*alb2a* and *Calb2b* are highly expressed in the CNS and peripheral nervous system, where they play a crucial role in regulating synaptic calcium concentration, thus contributing significantly to nervous system development. The combined loss of *Calb2a* and *Calb2b* leads to severe hydrocephalus, axial curvature defect, and yolk sac edema in zebrafish due to impaired neural tube folding and disorganized midbrain-hindbrain boundary [76]. *Atp1a3* (ATPase Na^+^/K^+^ transporting subunit α3) encodes an essential ion-transporting enzyme that regulates transmembrane Na^+^ and K^+^ gradients, playing a vital role in electrical excitation transmission of nerve and muscles. The *Atp1a3* knockdown in zebrafish can result in hydrocephalus due to disrupted transmembrane ion transport [78]. *Slc41a1* (solute carrier family 41 member 1) encodes Mg^2+^ transporter proteins located at the base membrane that participate in the transmembrane transport of Mg^2+^. Knockdown of *Slc41a1* with morpholino leads to body curvature, hydrocephalus, and kidney cysts in zebrafish as a result of disrupted intracellular Mg^2+^ homeostasis caused by blocked transmembrane Mg^2+^ transport [79].

### CNS-related genes

CH is not only a disorder of CSF dynamics, but also a brain disorder that leads to severe neurological impairment [80]. Most cells in the developing mammalian brain derive from the ventricular (VZ) and subventricular (SVZ) zones. The VZ consists of multipotent radial glia/NSCs, while the SVZ is composed of rapidly proliferating neural precursor cells (NPCs) [81]. These zones are crucial for neurodevelopment and any disruption, particularly within the VZ, can lead to stenosis or obliteration of the cerebral aqueduct of Sylvius, ultimately resulting in hydrocephalus [82–84]. This disturbance not only affects CSF flow but also simultaneously impairs the function of NSCs and ependymal cells, thereby linking hydrocephalus with abnormal neurogenesis [85–87]. Moreover, defects in membrane protein transporter-related genes could disrupt NSCs, leading to CH and associated cerebral malformations [1, 88–90]. Rodríguez et al. [82] proposed that gene mutations associated with cell junction proteins’ transport in NSCs could lead to the disruption of VZ, thereby resulting in aqueduct stenosis and hydrocephalus. NSCs play an important role in the growth of neurons and glial cells in the CNS [91, 92]. The dysfunction of NSC function hinders the polarity, proliferation, and differentiation of neurons. It is worth noting that NSC injury can also induce neurological disorders, such as cortical dysfunction, hydrocephalus, and periventricular heterotopia [91, 93]. Additionally, it is noteworthy that apoptosis within the CNS may impact neuronal development, resulting in hydrocephalus and nasal malformations [94]. In this section, we review 7 genes associated with CNS, whose dysfunction could contribute to hydrocephalus (Table 1) [82, 95–100].

*Pank2* (pantothenate kinase 2) encodes a protein belonging to the pantothenate kinase family and plays an essential role in cellular coenzyme A biosynthesis. *Pank2* morphant in zebrafish induced abnormal phenotypes including disrupted brain morphology, hydrocephalus, and edema in the heart region [95]. Downregulation of *Pank2* significantly impacts the development of neurons in the CNS and neuronal cells. *Ecrg4* (esophageal cancer-related gene 4) regulates the secretion of neuropeptides and is mainly expressed in the choroid plexus (CP) epithelial cells, brain ventricular, and central canal cells of the spinal cord. The product of *Ecrg4*, Augurin, contributes to the development of CNS and participates in the proliferation of NSC and NPC. Knockdown of *Ecrg4* using morpholino in zebrafish induced a hydrocephalus-like phenotype related to the damage of CNS [96].

### Subcommissural organ (SCO)-RF-related genes

RF, a network of threadlike glycoproteins suspended within the CSF, plays a pivotal role in the homeostatic regulation of the brain’s internal environment, by binding to and facilitating the transport and clearance of monoaminergic compounds. It is produced and released from the SCO of the brain, an active gland during development in most species including humans [101]. The SCO is an ependymal structure located at the roof of the third ventricle and the entrance to the mesencephalic aqueduct [102–104]. The RF extends through the SA, fourth ventricle, and central canal of the spinal cord to reach the caudal ampulla or fifth ventricle located at the end of the central canal [101]. Dysfunction of the SCO-RF complex is closely related to hydrocephalus phenotypes [103, 105]. Evidence suggested that the absence of RF or immunological damage to SCO could lead to stenosis or obliteration of cerebral aqueduct and defects in the neural canal (NCa), thereby impairing CSF circulation resulting in CH [106–108]. Moreover, the role of RF extends to neural development and axonal guidance, with its deficiency being associated with morphological brain defects, highlighting its multifaceted contribution to both normal physiology and disease pathology [88]. It is worth noting that RF is exclusively present in animals, except for humans. In humans, the secretory capacity of the SCO is robust in 3–5-month-old fetuses; however, it regresses significantly in 9-month fetuses. By 1-year-old, secretory ependymal cells shrink and cluster into islets interspersed with non-secretory cuboidal ependyma. This regression continues through childhood, limiting secretory parenchyma to scattered islets by the ninth year. Despite the absence of RF in humans, SCO-spondin, the unpolymerized form of RF, is present and soluble in CSF, thus impacting brain development [109]. It also participates in certain aspects of neurogenesis, such as the cell cycle of NSCs, neuronal differentiation, and axon pathfinding [104]. In this section, we discuss 2 genes linked to RF function that contribute to the development of hydrocephalus (Table 1) [105, 110].

*Camal* encodes a protein associated with cell adhesion. *Camel* regulates the development of brain ventricular, and loss of camel function in zebrafish leads to the manifestation of hydrocephalus and scoliosis. Deletion of camel has been shown to result in hydrocephalus due to defects in RF synthesis, resulting from abnormal CSF flow [105]. *Msx1* (Msh homeobox 1) is involved in regulating DNA-binding transcription factor activity and is widely expressed in neuroepithelial cells. *Msx1* mutants exhibit severe hydrocephalus at birth, accompanied by abnormal SCO development. Additionally, RF was found to be absent in *Msx1* mutant mice [110]. This suggests that *Msx1* mutants inhibit RF synthesis by affecting normal SCO development, thereby affecting CSF flow.

### Others

Table 1 also highlights five additional genes and small molecular substances linked to hydrocephalus [111–115]. However, the mechanisms by which these genes influence the progression of hydrocephalus are not fully understood or categorized as mentioned earlier. Furthermore, the inflammatory/immune response may also be associated with the progression and severity of hydrocephalus [2, 116]. In the *hyh* mice and *HTx* rats (two animal models of fetal-onset hydrocephalus), the onset of ventricle disruption is correlated with the infiltration of macrophages and lymphocytes into denuded.

The expression of *β-Pix* [Rho guanine nucleotide exchange factor (GEF) 7b] is widespread in both the brain and blood vessels, where it plays a role in regulating cerebral vascular stability. In zebrafish, mutation of the *β-Pix* gene can lead to obvious hydrocephalus and severe intracranial hemorrhage during early embryonic development. It has been hypothesized that deleting *β-Pix* may disrupt vascular stability, potentially affecting CSF circulation [112]. Thioredoxin1 is an antioxidant protein with reactive oxygen species (ROS) scavenging capabilities that govern processes such as cell proliferation, migration, apoptosis, and inflammation. Zebrafish injected with thioredoxin1 morpholine exhibit hydrocephalus and midbrain malformations [115]. Deletion of *thioredoxin1* triggers a significant increase in ventricular epithelial cell apoptosis while disrupting vascular endothelial cell migration, ultimately leading to hydrocephalus.

## Discussion

In this review, we have comprehensively summarized the genetic factors and molecular mechanisms of CH in both human subjects and animal models. The results from human sequencing and validated genes showed that these genes are related to dysfunction of the central system, impaired cilia movement, and abnormalities in SA. By utilizing animal models such as mice and zebrafish, it becomes feasible to further investigate additional genes related to hydrocephalus pathology. These genes can be systematically classified into 4 principal groups: those linked to ciliary function, ion transport, CNS function, and RF synthesis. Genes related to ciliary function play an important role in regulating the synthesis, formation, and movement of cilia, which is closely connected with CSF absorption. Ion transporter-related genes primarily disrupt homeostasis by dysregulating the ions’ transportation processes, thus impacting CSF secretion. Mutation in CNS function-related genes predominantly affects the development, function, and apoptosis of nerve cells, which might result in potential disturbances in brain morphology. Additionally, the RF synthesis-related genes dysregulate the formation and morphology of NCa, influencing CSF circulation. The identification of these genes in CH animal models provides valuable resources for validation within larger clinical cohorts of CH patients.

Genetic insights hold profound significance in the management of CH. The pathogenesis of this complex disease may be closely linked to multiple gene variants. Genetic research aids in identifying these key gene variants, thereby unraveling the underlying mechanisms of the disease and paving the way for innovative treatment approaches [117]. For instance, if a specific genetic variant is found to be intricately associated with the disease, gene-editing techniques or gene therapy can be employed to correct this variant, ultimately aiming to cure the condition [118–120]. Furthermore, genetic understanding promotes personalized healthcare. As each individual has a unique genome, responses to illnesses and treatment outcomes naturally differ. Genetic research enables tailored therapies based on a patient’s genotype, optimizing treatment efficiency and minimizing adverse effects [121]. Moreover, genetic insights facilitate more accurate disease prediction and risk assessment. Genetic screenings allow us to anticipate an individual’s susceptibility to certain illnesses, enabling proactive preventive measures. This prediction is particularly crucial for genetic conditions such as CH. In conclusion, genetic insights offer immense potential to revolutionize disease treatment. As genetic research advances and technology evolves, we are poised to deliver more precise and effective medical care in the foreseeable future. Nevertheless, it is crucial to recognize that genetics do not hold all the answers, they address only a portion of health conditions. Hence, a holistic approach encompassing genetics as well as environment and lifestyle factors is essential for devising comprehensive treatment plans.

Addressing the complexities associated with CH necessitates developing a multimodal detection approach that integrates both clinical observations along radiological phenotypic characteristics alongside genotypic analysis for effective implementation within a clinical setting. This comprehensive approach plays a pivotal role in augmenting diagnostic precision and specificity, crucial when dealing with conditions where initial symptoms may not manifest at birth but evolve gradually over time. The utilization of clinical radiological medical imaging technology like CT as well as MRI offers substantial benefits, particularly in identifying structural anomalies within the brain including aqueduct stenosis, Dandy-Walker malformation, arachnoid cysts, and neural tube defects. Additionally, the insight provided by genotype data facilitates a deeper understanding of the onset and progression mechanisms related to CH pathology. However, it should be noted that genetic analysis alone may have limitations when elucidating complex presentations involving skull morphology, extracranial structures, and skeletal deformities. Therefore, a synergistic amalgamation encompassing genotype data along with detailed examination through clinical and radiologic means holds promise for expediting precise disease identification. Zhang et al. [122] integrated key findings from their study which involved combining patient-specific traits, and molecular analyses via neuroimaging modalities such as MRI/CT scans, gene mutation tests, and metabolic assessments. Moreover, Rijken et al. [123] demonstrated how 3D-CT reconstruction technology played an indispensable role in delineating morphometric changes in foramen magnum configuration as well as the presence of ventriculomegaly among pediatric patients diagnosed with craniosynostosis; this technique exhibits considerable potential for facilitating CH diagnostics. Consequently, this multimodal detection strategy, involving integration between radiologically derived phenotypes and genotype analytics, serves not only to enhance diagnostic precision and treatment efficacy but also paves the way for tailored medical interventions catering to individual patient needs. With ongoing advancements in technology-driven genomic research coupled with expanding horizons within clinical applications, it is anticipated that future management strategies will enable more accurate and effective treatment across diverse spectrums of ailments.

The phenotypic manifestations of genetic defects are remarkably diverse and complex. Pathogenic genes associated with hydrocephalus may also present in other tissues or organs, leading to a range of comorbidities. For instance, dysfunctional ciliary genes can also trigger renal cysts and scoliosis [46, 48, 49, 54, 56, 58]. Additionally, the loss of function of the *SLC25A4* gene can lead to severe cardiomyopathy, scoliosis, cataracts, and depression [124]. Understanding the associated complications of hydrocephalus is essential for identifying the underlying pathology and implementing personalized treatment. If patients exhibit symptoms of hydrocephalus, early intervention, and targeted treatments should be provided to prevent associated comorbidities.

Further research into the genetic and pathogenesis of CH will facilitate the development of animal models for investigating drug treatment options. Currently, the field of drug therapy for CH remains largely unexplored, and establishing effective animal models of hydrocephalus provides a platform for exploring potential drug targets.

## Conclusions

In this review, we have provided a comprehensive summary of recent discoveries regarding the genetic targets of CH in both human and animal models. In addition to the 4 confirmed genes associated with CH (X-linked genes *L1CAM* and *AP1S2*, autosomal recessive *MPDZ*, and *CCDC88C*). We have also reviewed 35 genes identified through gene sequencing in human cases, as well as numerous related genes in the CH animal model. These findings warrant further validation through extensive clinical studies involving a large cohort of CH patients. The implicated genes primarily participate in 4 pathways and may contribute to comorbidities affecting other organ functions where these related genes are expressed.

## Data Availability

Not applicable.

## Abbreviations

AP1S2: Adaptor-related protein complex 1 subunit sigma 2
AK9: Adenylate kinase 9
CCDC88C: Coiled-coil domain-containing 88C
CC2D2A: Coiled-coil and C2 domain containing 2A
CH: Congenital hydrocephalus
CNS: Central nervous system
CP: Choroid plexus
CRB2: Crumbs cell polarity complex component 2
CSF: Cerebral spinal fluid
CWH43: Cell wall biogenesis 43 C-terminal homolog
dcLNs: Deep cervical lymphatic nodes
EC: Ependymal cell
GPI: Glucose-6-phosphate isomerase
IDA: Inner dynein arms
iNPH: Idiopathic normal pressure hydrocephalus
ISF: Interstitial fluid
L1CAM: L1 cell adhesion molecule
mLVs: Meningeal lymphatic vessels
MMACHC: Metabolism of cobalamin-associated C
MPDZ: Multiple PDZ domain crumbs cell polarity complex component
NCa: Neural canal
NSPC: Neural stem/progenitor cells
NSC: Neural stem cell
NPC: Neural progenitor cell
ODA: Outer dynein arms
PCD: Primary ciliary dyskinesia
PCP: Planar cell polarity
PTCH1: Patched 1
RF: Reissner’s fiber
RGC: Radial glial cells
SA: Sylvius aqueduct
SCO: Subcommissural organ
SMARCC1: SWI/SNF related, matrix associated, actin dependent regulator of chromatin subfamily C member 1
SVZ: Subventricular zone
TRIM71: Tripartite motif containing 71
VZ: Ventricular zone
WES: Whole exome sequencing
